# Vitamin D Nutritional Status in the Middle East and North Africa Region: A Systematic Review and Meta-analysis

**DOI:** 10.1016/j.cdnut.2025.107577

**Published:** 2025-10-21

**Authors:** François Machuron, Robin Dessalles, Léa Ribet, Hayat Bentouhami, Joséphine Escutnaire, Nele Brusselaers, Mickael Durand-Dubief

**Affiliations:** 1 Lesaffre Institute of Science & Technology, Lesaffre, Marcq-en-Barœul, France; 2 Nutrition & Health, Robin Dessalles EI, Lyon, France; 3 Department of Family Medicine and Population Health, University of Antwerp, Antwerp, Belgium; 4 Gnosis Research and Development Department, Gnosis by Lesaffre, Lesaffre, Marcq-en-Baroeul, France; 5 Department of Women's and Children's Health, Karolinska Institutet, Stockholm, Sweden; 6 Department of Public Health and Primary Care, Ghent University, Ghent, Belgium

**Keywords:** dietary fortification, Middle East and North Africa (MENA), public health strategies, serum 25-hydroxyvitamin D [25(OH)D], gender differences, vitamin D deficiency

## Abstract

**Background:**

Adequate vitamin D status is crucial for health, especially for skeletal health. The prevention of vitamin D deficiency disorders represents a major public health challenge in countries with low sunlight exposure. Nonetheless, several studies conducted in the Middle East and North Africa (MENA) region have reported surprisingly high prevalences of vitamin D deficiency, despite high sunlight availability. Even in those countries, dietary and lifestyle recommendations, including nutritional fortification policies, may be needed.

**Objectives:**

The present study aimed to systematically update and assess the vitamin D status in the MENA region using a meta-analysis approach.

**Methods:**

This meta-analysis included 41 studies (2000–2022) evaluating serum vitamin D levels in healthy MENA adult populations, using pooled analyses and meta-regressions to assess the overall levels, sex differences, and the impact of covariates such as age and body mass index (BMI). Some studies identified as source of heterogeneity were excluded, and random-effects models were used in meta-analyses to control variability of effect sizes. Registration ID https://doi.org/10.17605/OSF.IO/FK4WM.

**Results:**

High variability from country to country was observed in vitamin D status in the MENA region, ranging from 9 ± 7 to 88 ± 76 nmol/L. The overall mean serum vitamin D concentration was 46 (95% confidence interval: 40, 52) nmol/L (below the recommended cutoff of 50 nmol/L), with a 4 nmol/L higher level in men compared with women, and no significant impact of age or BMI.

**Conclusions:**

This meta-analysis provides a comprehensive assessment of vitamin D status in adults from the MENA region, highlighting the widespread vitamin D deficiency in the region, with potential gender differences, but no clear impact of age or BMI—findings that could reinforce targeted, regional public health strategies to improve vitamin D status through lifestyle and nutrition.

## Graphical abstract

**Figure:**
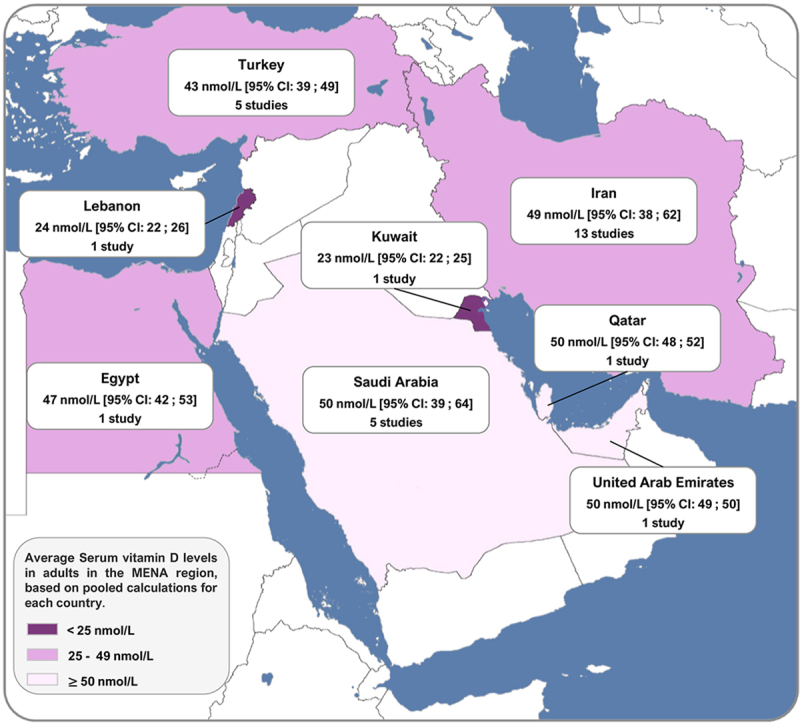

## Introduction

Vitamin D is a crucial component for musculoskeletal health. Through its ability to regulate calcium and phosphorus levels in the body, it plays a key role in bone homeostasis, signal transduction, and neuromuscular function [1,2]. It is also known for its action as an immunomodulator [3]. Low serum vitamin D has been associated with an increased loss of bone mineral density and bone mineral content, an increased risk of fractures and falls, muscle weakness and osteomalacia in adults [1,2]. In children, vitamin D deficiency typically leads to rickets [4]. Associations have long been reported between vitamin D status and a range of noncommunicable diseases, including metabolic syndrome [5,6], type 2 diabetes [7], hypertension, and autoimmune diseases [8]. In women also, vitamin D status has also been associated with miscarriage [9] and fertility [10]. Owing to the key role of vitamin D in the maintenance of health, it is unambiguous that achieving sufficient serum levels within populations is of public health importance.

Despite being called a fat-soluble vitamin, vitamin D is more a prohormone than a vitamin or a nutrient, as endogenous production occurs in the skin when the precursor of vitamin D3 is activated in response to UVB radiation [2,3,11,12]. Therefore, vitamin D requirements in humans could, in theory, be entirely met through exposure to sunlight. In practice, however, dietary sources of vitamin D (limited to fish, meat, or eggs for conventional foods, i.e., not fortified) [13] are of importance as sunlight exposure may be difficult to achieve, especially in winter in higher-latitude countries [2,11,14]. These characteristics led to the development of fortification and supplementation strategies in those countries [11,15], whereas until recently, it was assumed that these policies were unnecessary in countries with abundant sunlight all year round. However, a growing body of evidence has reported concerning prevalences of vitamin D deficiency worldwide [12,16], even in geographical areas with high sunlight availability, such as Africa [17] or the Middle East [[18], [19], [20]]. Deficiency in those countries has even been reported to be higher than in Northern and Western Europe [18]. Over recent years, a growing number of population-based studies assessed the prevalence of vitamin D deficiency in the Middle East and North Africa (MENA) regions. Variability between studies in population characteristics, measurement methods, and deficiency thresholds has been reported [21], making it sometimes difficult to draw a clear picture of the extent to which the local population suffers from vitamin D deficiency. As it seems recent meta-analyses on the topic focused on specific countries or populations [[22], [23], [24], [25]], we aim to provide an updated systematic review and meta-analysis of population-based studies focusing on the MENA regions to assess serum vitamin D and estimate the level of deficiency.

## Methods

The present work followed the PRISMA guideline [26]. The primary outcome investigated for this meta-analysis was the average serum 25-hydroxyvitamin D [25(OH)D], as it is acknowledged as the best indicator of vitamin D status [2,18]. Additionally, focusing on serum vitamin D rather than percentage of deficiency enabled to avoid issues related to the variety of thresholds used for defining vitamin D deficiency across the literature. The study was registered in the Open Science Framework registry (https://doi.org/10.17605/OSF.IO/FK4WM)

Scientific articles were searched using 3 different databases: PubMed, Cochrane Library, and The Lens. Queries were created relevant to each database, using keywords related to vitamin D (vitamin D, cholecalciferol, 25-hydroxyvitamin), nutritional status (deficiency, sufficiency, insufficiency, hypovitaminosis), study design (population, survey, observational, cross-sectional, cohort), and geographical indicators ("Middle East," Arabia, "Gulf countries," Africa). The full search strings are available in Supplementary Table 1. Searches were last updated in June 2024.

The selected articles had to be: peer-reviewed articles, of any date, available in English, cross-sectional or prospective population-based studies, conducted on healthy adults, in the MENA region as defined by the United Nations [27], with at least mean serum 25(OH)D available (in nmol/L or ng/ml) for the whole sample.

Articles were excluded if they were case-control studies, studies with a sample size of <100 individuals, studies conducted on pregnant women, studies on samples of the population with very limited representativity (e.g., students or care facility personnel), or studies conducted on institutionalized people.

Two researchers screened records independently to determine their compliance with eligibility criteria. Publications data were collected using a specific electronic form. Risk of bias analysis was performed using a modified version of the JBI critical appraisal tool for prevalence studies [28]. A general appreciation of risk of bias of a specific study was given based on whether doubt arose from answers to 1 of the 8 items proposed by the tool.

Information on measurement methods was extracted when available. Other information included any parameters known to be potentially associated with vitamin D status, such as: age, sex, BMI (in kg/m^2^), level of physical activity, rural or urban residence, diet, occupation, education, clothing, or supplements intake.

### Statistics

For the meta-analysis, mean serum vitamin D was expressed in nmol/L, with 95% confidence interval (CI). Values expressed in ng/mL were converted (1 ng/mL = 2.5 nmol/L). Studies only mentioning median and IQR were excluded from the analysis. The meta-analysis was performed using R software meta package. Random-effect models were used to control the high heterogeneity of studies. Sources of heterogeneity were identified using both Funnel and Baujat plots. Studies identified as major sources of heterogeneity have been iteratively removed from the analysis. I^2^ metric for the assessment of subgroup and overall heterogeneity has been used (no heterogeneity: 0%–25%; low heterogeneity: 25%–50%; substantial heterogeneity: 50%–75%; considerable heterogeneity: 75%–100%). Subgroup analyses (by age, sex, BMI, or study risk of bias) were performed using metaregression.

## Results

**FIGURE 1:**
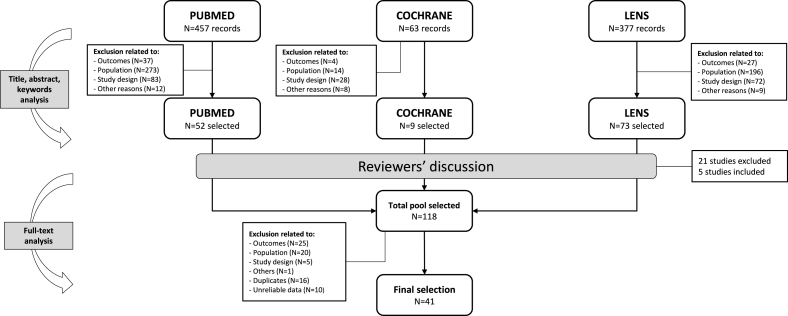
Detailed flow-chart of the study selection process.

A total of 897 records were screened, of which 118 complied with eligibility criteria after discussion between reviewers. Full-text analysis led to further exclusions, and after removing duplicates and studies with unreliable data, the final selection included 41 articles, reporting 41 studies. The PRISMA flow diagram (Figure 1) details the selection process with reasons for exclusion.

Study characteristics are summarized in Table 1 [[29], [30], [31], [32], [33], [34], [35], [36], [37], [38], [39], [40], [41], [42], [43], [44], [45], [46], [47], [48], [49], [50], [51], [52], [53], [54], [55], [56], [57], [58], [59], [60], [61], [62], [63], [64], [65], [66], [67], [68], [69]]; additional details on the study characteristics are provided Supplementary Table 2.

**TABLE 1:** Characteristics of the included studies.

Reference	Year	Country	Design	Target population	*N*	Gender (F/M)	Mean age (y)	Age range (y)	Mean BMI (kg/m^2^)	Serum 25(OH)D measurement method	Mean serum 25(OH)D (nmol/L)	Risk of bias	
Al-Daghri et al. [29] (2018)	2018	KSA	Cross-sectional	Adults	711 (w/o asthma)	387/324	27.1 ± 0.5	18–50	26.2 ± 0.2	ECLIA	38 ± 1	39%	Medium
Al-Daghri et al. [30] (2015)	2015	KSA	Cross-sectional	Children & adults	565	316/249	F: 32.2 ± 0.6 M: 27.9 ± 0.8 *p* < 0.001	NM	28.6 ± 0.4	ECLIA	36 ± 1	39%	Medium
AlFaris et al. [31] (2019)	2019	KSA	Cross-sectional	Adult women	166	100%/0%	NM	30–65	NM	RIA	52 ± 33	61%	Medium
Alfhili et al. [32] (2022)	2022	KSA	Cross-sectional	All	14,229	8274/5955	NM	3–110	NM	NM	17 ± 0	28%	Low
Alkhenizan et al. [33] (2017)	2017	KSA	Cross-sectional	Adults	1723	1452/271	NM	NM	NM	NM	48 ± 37	56%	Medium
AlQuaiz et al. [34] (2020)	2020	KSA	Cross-sectional	Adults	1717	1064/653	39.5 ± 9.0	30–75	30.0 ± 6.3	ECLIA	33 ± 19	17%	Low
AlQuaiz et al. [35] (2019)	2019	KSA	Cross-sectional	Adults	2131	1285/846	F: 42.1 ± 10.1 M: 42.7 ± 11.5	NM	NM	ECLIA	32 ± 16	17%	Low
Alsejari [36] (2018)	2018	Kuwait	Cross-sectional	Adults	276	146/130	31.55 ± 13.52	NM	NM	NM	23 ± 11	72%	High
Alsuwadia et al. [37] (2013)	2013	KSA	Cross-sectional	Adults	488	243/245	37.43 ± 11.32	18–72	29.25 ± 5.72	ECLIA	71 ± 33	39%	Medium
Ardeshir Larijani et al. [38] (2014)	2014	Iran	Cross-sectional	Adults	2451	1337/1114	All: 42.43 ± 13.9 F: 41.8 ± 13.0 M: 42.9 ± 14.9	20–70	26.6 ± 4.7	RIA	87 ± 69	11%	Low
Ehrampoush et al. [39] (2021)	2021	Iran	Cross-sectional	Adults	2160	1057/1103	39.8 ± 10.8	NM	NM	ELISA	23 ± 9	33%	Medium
Farhud et al. [40] (2019)	2019	Iran	Descriptive	Whole population	308,005	217,823/85,028 72.3%/27.7%	39.08 ± 19.87	NM	NM	NM	64 ± 55	50%	Medium
Gannagé-Yared et al. [41] (2000)	2000	Lebanon	Cross-sectional	Adults	316	217/99	All: 40.0 ± 5.6 F: 39.4 ± 5.6 M: 41.3 ± 5.5	30–50	25.9 ± 4.1	RIA	24 ± 18	44%	Medium
Gerber et al. [42] (2016)	2016	Qatar	Cross-sectional	Adult women	523	100%/0%	49.7 ± 5.5	40–60	34.5 ± 6.0	RIA	50 ± 23	28%	Low
Gerges et al. [43] (2021)	2021	Egypt	Cross-sectional	Adult women	100	100%/0%	34 ± 8.47	19–49	32.48 ± 6.57	EIA	47 ± 28	39%	Medium
Göktaş et al. [44] (2020)	2020	Turkey	Cross-sectional	Adults	11,734	9142/2592	46.5 ± 16.9	NM	NM	ECLIA	42 ± 29	22%	Low
Golbahar et al. [45] (2013)	2013	Bahrain	Cross-sectional	Adults	421	208/213	NM	18–60	NM	LC-MS	28 ± 17	44%	Medium
Hataysal et al. [46] (2019)	2019	Turkey	Cross-sectional	Adults	2007	1526/481	NM	18–65	NM	LC-MS	42 ± 27	39%	Medium
Hekimsoy et al. [47] (2010)	2010	Turkey	Cross-sectional	Adults	391	272/119	45.11 ± 17.28	NM	NM	HPLC	42 ± 33	17%	Low
Hossein-Nezhad et al. [48] (2015)	2015	Iran	Cross-sectional	Adults	646	486/160	47.62 ± 14.46	20–79	26.65±4.84	RIA	31 ± 21	17%	Low
Hosseinpanah et al. [49] (2008)	2008	Iran	Cross-sectional	Adult women	245	100%/0%	57.7 ± 7.0	40–80 Median (IQR): 57 (53–62)	29.6 ± 4.9	RIA	73 ± 62	17%	Low
Isgin-Atici et al. [50] (2022)	2022	Turkey	Cross-sectional	Adults	396	NM	NM	24–50	NM	ELISA	62 ± 4	39%	Medium
Kader et al. [51] (2019)	2019	Turkey	Cross-sectional	Adults	6774	5111/1663	NM	NM	NM	CLIA	39 ± 33	56%	Medium
Kaykhaei et al. [52] (2011)	2011	Iran	Cross-sectional	Adults	993	562/431	36.71 ± 14.28	20–88	24.64 ± 4.98	CLIA	34 ± 29	44%	Medium
Khashayar et al. [53] (2011)	2011	Iran	Cross-sectional	Adults	3764	2211/1553	42.31 ± 13.63	19–76	NM	RIA	88 ± 76	28%	Low
Khashayar et al. [54] (2014)	2014	Iran	Cross-sectional	Adults	3669	2116/1553	42.08 ± 13.56	NM	26.6 ± 4.8	RIA	86 ± 60	17%	Low
Khosravi-Boroujeni et al. [55] (2017)	2017	Iran	Longitudinal	Adults	370	154/216	NM	NM	NM	ELISA	56 ± 45	22%	Low
Masoompour et al. [56] (2008)	2008	Iran	Cross-sectional	Adult men	520	0%/100%	45 ± 15	NM	26.2±1.6	RIA	35 ± 17	22%	Low
Mehboobali et al. [57] (2015)	2015	Pakistan	Cross-sectional	Adults	858	507/351	32.5 ± 10.7	18–60	NM	ECLIA	52 ± 18	44%	Medium
Mohammadzadeh et al. [58] (2020)	2020	Iran	Cross-sectional	Adults	100	61/39	37 ± 13	20–50	23.00 ± 3.25	ECLIA	42	50%	Medium
Moini et al. [59] (2015)	2015	Iran	Cross-sectional	Adult women	117	100%/0%	30.82 ± 7.12	NM	24.41 ± 3.88	ELISA	9 ± 7	44%	Medium
Naeem et al. [60] (2011)	2011	KSA	Cross-sectional	Adults	180	97/83	40.8	19–72	NM	EIA	69	39%	Medium
Nikooyeh et al. [61] (2017)	2017	Iran	Cross-sectional	Adults	1406	751/655	38.4 ± 8.5	NM	27.1 + 4.7	EIA	27 ± 18	11%	Low
Nikooyeh et al. [62] (2021)	2021	Iran	Cross-sectional	Adults	1111	614/497	38.8 ± 8.1	19–65	Summer: 26.7 ± 4.6 Winter: 27.2 ± 4.7	EIA	35 ± 25	17%	Low
Omrani et al. [63] (2006)	2006	Iran	Cross-sectional	Adult women	676	100%/0%	42.3 ± 13.4	20–74	26.4 ± 1.9	IRA	29 ± 23	22%	Low
Pourhashem et al. [64] (2012)	2012	Iran	Cross-sectional	Elderly	193	88/105	68.39 ± 6.17	60–88	NM	ELISA	51 ± 42	44%	Medium
Saad et al. [65] (2020)	2020	Lebanon	Cross-sectional	All	Total: 151,705 Children: 9574 Adults: 103,653 Elderly: 38,478	46,099/96,032	Children: 11 ± 5 y Adults: 44 ± 13 y Elderly: 74 ± 6 y	NM	NM	RIA/CLIA/ECLIA	60 ± 44	56%	Medium
Sadat-Ali et al. [66] (2014)	2014	KSA	Cross-sectional	Adults	200	150/50	45.7 ± 16.1	NM	NM	LC-MS/MS	54 ± 34	39%	Medium
Safari et al. [67] (2017)	2017	Iran	Cross-sectional	Adults	210	146/64	35.8 ± 10.8	20–70	27.15±5.33	ELISA	59	22%	Low
Yammine and Al Adham [68] (2016)	2016	UAE	Cross-sectional	All	7924	5506/2418	37	NM	NM	IMF	50 ± 28	33%	Medium
Yeşiltepe-Mutlu et al. [69] (2020)	2020	Turkey	Cross-sectional	All	108,742	79,293/29,449	NM	NM	NM	LC-MS	54 ± 33	39%	Medium

Abbreviations: 25(OH)D, 25-hydroxyvitamin D; ECLIA, electrochemiluminescence immunoassay; EIA, enzyme immunoassay; IMF, immunofluorescence; CLIA, chemiluminescence immunoassay; KSA, Kingdom of Saudi Arabia; LC-MS/MS, liquid chromatography-mass spectrometry; NM, not mentioned; RIA, Radioimmunoassay; UAE, United Arab Emirates.

Most studies selected were conducted in Iran (*N* = 17), Saudi Arabia (KSA; *N*=10), and Turkey (*N* = 6). Two studies were performed in Lebanon. The remaining 6 studies focused on populations from Bahrain, Egypt, Kuwait, Pakistan, Qatar, and the United Arab Emirates (UAE) (Supplementary Table 3). The 41 studies represented a total pool of 594,712 individuals, aged between 18 and 110 y. Fifty-six percent (56%) of studies were classified as with a medium risk of bias, 42% with low risk, and 2% with high risk.

**FIGURE 2:**
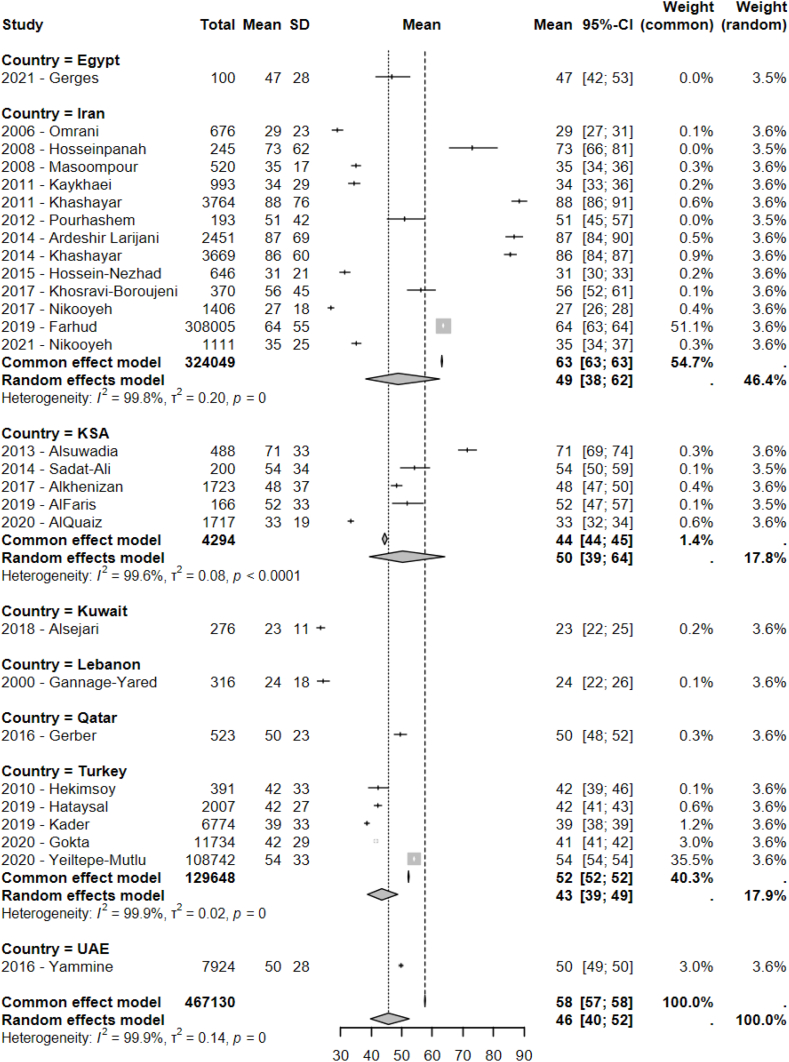
Forest plot of the pooled average serum vitamin D from included studies conducted in the Middle East and North Africa (MENA) regions (*N* = 28), sorted by country. CI, confidence interval; KSA, Kingdom of Saudi Arabia; UAE, United Arab Emirates.

After removing studies with insufficient variability, excessive weight (*N* = 4) [29,39,50,59] and missing values (*N* = 9) [30,32,35,45,57,58,60,65,67], average serum 25(OH)D of the whole population sample from the 28 retained studies [31,33,34,[36], [37], [38],[40], [41], [42], [43], [44],[46], [47], [48], [49],[51], [52], [53], [54], [55], [56],[61], [62], [63], [64],^66^,^68^,^69^] was 46 nmol/L (95% CI: 40, 52). Average serum vitamin D by country was 49 nmol/L (95% CI: 38, 62) in Iran (*N* = 13) [38,40,48,49,[52], [53], [54], [55], [56],[61], [62], [63], [64]], 50 nmol/L (95% CI: 39, 64) in KSA (*N* = 5) [[31], [32], [33], [34],^37^,^66^], and 43 nmol/L (95% CI: 39, 49) in Turkey (*N* = 5) [44,46,47,51,69]. The analysis for the remaining countries, based on 1 study each, revealed an average serum 25(OH)D of 47 nmol/L (95% CI: 42, 53) in Egypt [43], 23 nmol/L (95% CI: 22, 25) in Kuwait [36], 24 nmol/L (95% CI: 22, 26) in Lebanon [41], 50 nmol/L (95% CI: 48, 52) in Qatar [42], and 50 nmol/L (95% CI: 40, 52) in UAE [68] (Figure 2).

On 30 studies with prevalence values available, comprising 124,897 patients, we obtained an estimate of a global prevalence of insufficient vitamin D concentration [25(OH)D < 50 nmol/L] of 42.8%.

**FIGURE 3:**
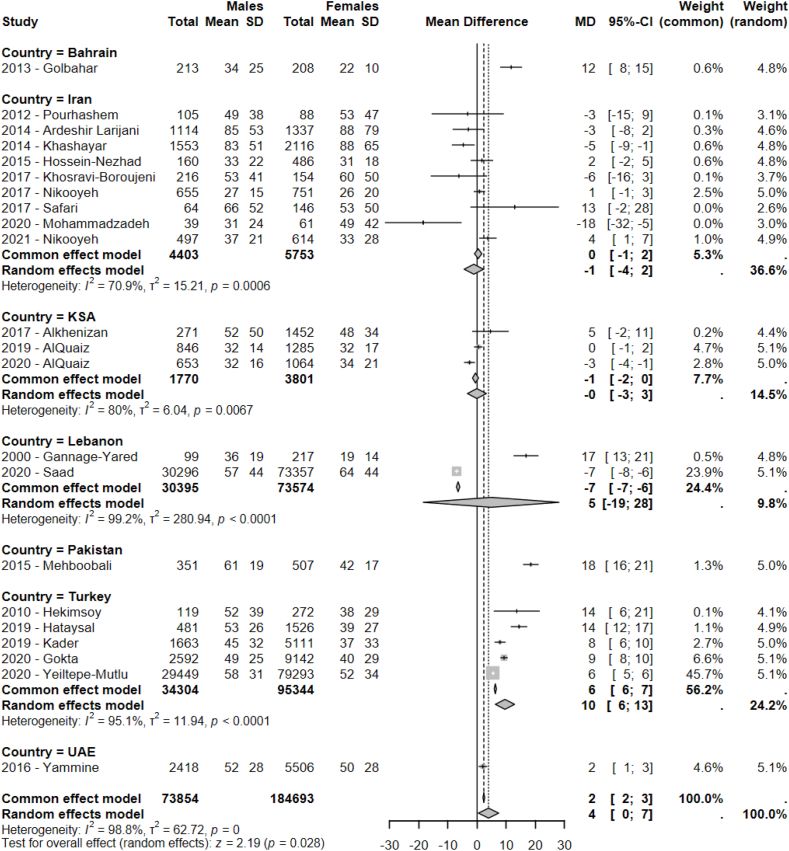
Forest plot of the difference between sex in pooled average serum vitamin D from included studies conducted in the Middle East and North Africa (MENA) regions (*N* = 22). CI, confidence interval; KSA, Kingdom of Saudi Arabia; UAE, United Arab Emirates.

**FIGURE 4:**
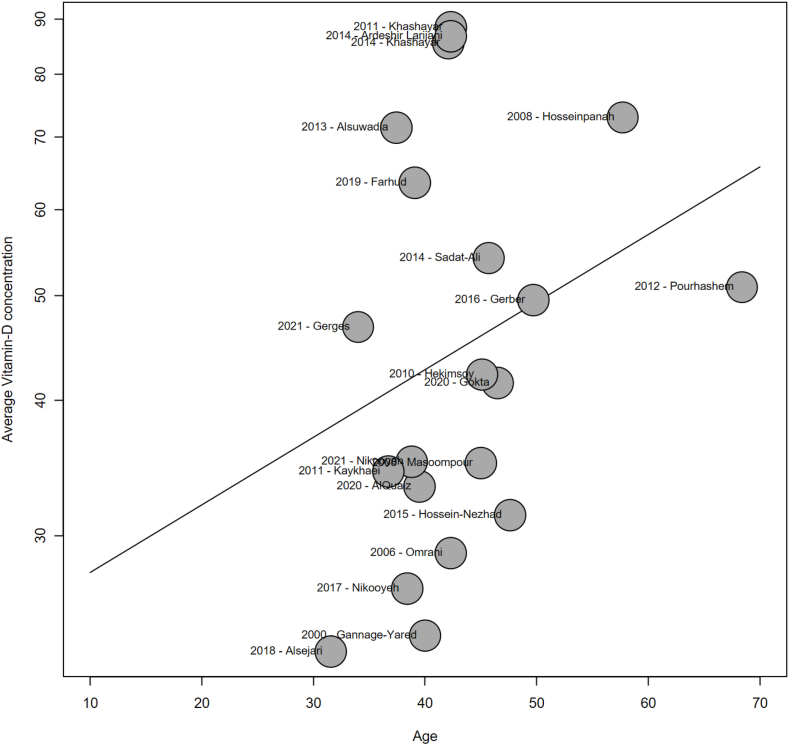
Metaregression: impact of the age on pooled serum vitamin D levels from included studies conducted in the Middle East and North Africa (MENA) regions (*N* = 21) (*P* = 0.218).

Analysis by sex revealed a significantly lower average serum vitamin D in women compared with men (*P* = 0.028), with an average difference of 4 nmol/L between groups (95% CI: 0, 7), based on 22 studies (Figure 3) [[33], [34], [35],^38^,^41^,[44], [45], [46], [47], [48],^51^,^54^,^55^,^57^,^58^,^61^,^62^,^64^,^65^,[67], [68], [69]]. This difference was more pronounced in Turkey (*N* = 5) [44,46,47,51,69], Bahrain (*N* = 1) [45], and Pakistan (*N* = 1) [57], where men had average serum vitamin D levels 10, 12, and 18 nmol/L higher than women, respectively. The effect of age on vitamin D status was also investigated (Figure 4). No significant effect of age on serum 25(OH)D could be identified (*P* = 0.22). Similarly, no significant effect of BMI on serum vitamin D was observed (*P* = 0.33) (Supplementary Figures).

## Discussion

The present work aimed at performing a meta-analysis of studies assessing the vitamin D status of adult populations in the MENA region. The pooled average level of serum vitamin D in this region was 46 nmol/L, consistent with other systematic reviews reporting serum 25(OH)D in the region between 25 and 50 nmol/L [18]. Thresholds for vitamin D sufficiency are still subject to debate, with institutions such as the European Food Safety Authority or the European Calcified Tissue Society establishing a threshold of 50 nmol/L for sufficiency, whereas the Endocrine Society considered sufficiency only above 75 nmol/L [18]. Conversely, AlQuaiz et al. [35] proposed, based on parathyroid hormone secretion, a cutoff of 30 nmol/L for the Saudi population specifically, under which no specific treatment or follow-up is recommended. Prospective population-based studies assessing the long-term suitability of such a threshold as regards health outcomes in local populations are still lacking. In the meantime, guidelines from the Gulf Cooperative Council (GCC) countries or from KSA also adopted the 50 nmol/L value as the deficiency cutoff [70,71].

Whatever the threshold used, our results seem to confirm that vitamin D sufficiency should not be taken for granted in countries with high sunlight availability, especially in women, as indicated by the significantly lower average serum vitamin D identified in women compared with men. This is in line with other publications across the literature [12,20,72,73]. Deficiency rates in the MENA region may be explained by a range of different factors mainly preventing sun exposure and thus endogenous synthesis of vitamin D. These factors may be physiological, such as skin pigmentation, but seem mostly related to lifestyle and cultural habits [72]. Among these, whole-body clothing was significantly associated with vitamin D deficiency in a range of individual studies [36,74,75]. One study reported whole-body clothing as a risk factor for low serum vitamin D in women, but not in men [41], which led to the assumption that different clothing and lifestyle habits in women compared with men could be an explanation for serum vitamin D differences. However, other studies reported a similar effect of whole-body clothing on serum vitamin D in both genders [47]. Of note, our study did not identify gender differences in serum vitamin D in Iran or KSA, which may indicate that the situation is setting dependent, though more studies from other countries in the same region are needed to better inform on this matter.

Contrary to Western or Nordic countries, seasonal variation in vitamin D status would be less expected in the MENA region owing to all-year-round sunlight availability and long summer seasons. However, significantly higher levels of serum vitamin D in summer compared with winter have been commonly reported [46,65,69,76,77], which may add to the heterogeneity observed in studies. In our study, no significant association between serum 25(OH)D and age could be noted. This is surprising as the skin’s capacity to produce vitamin D is known to decrease with age [2,78]. The fact that most study populations in our selection of studies had relatively similar mean age (around 40 y) may have limited the possibility of detecting significant differences in serum 25(OH)D between age groups. Nevertheless, an increased risk of vitamin D deficiency in younger compared with older age groups has been reported in several individual studies [31,36,37,52,67]. This may be due to other lifestyle factors associated with increased age, such as increased supplementation [34], or more time spent outdoor [76], though differences between genders were also noted for the latter point. Indeed, 1 study reported that elderly women may not benefit from sun exposure as much as men, owing to a more home-based sedentary lifestyle [77]. More time spent outdoors was also proposed as an explanation of the higher serum vitamin D values observed in children compared with adults in several studies [12,36,69]. Accordingly, working in an outdoor environment has been associated with significantly less vitamin D deficiency compared with working indoors [36,74] Also, the location of residence may have an impact on vitamin D status. In the present study, we attempted a comparison between the average serum vitamin D of populations living in urban compared with rural areas, but data from rural areas only could not be isolated. Though no significant association between area of residence and serum vitamin D was reported in Iranian adults [55], another study reported a more than 2-fold increased risk of having vitamin D deficiency or insufficiency in Turkish adults residing in urban areas compared with those living in semiurban areas. In the same study, the risk of having vitamin D insufficiency or deficiency when living in rural areas was 4 times higher than when living in semiurban areas , though the difference was not significant [47]. An older study also reported a significant positive effect of rural dwelling on serum vitamin D levels [41]. Urban lifestyle, which includes working within closed doors, living in an apartment without a garden, or having access to less outdoor activities, may decrease exposure to sunlight and thus serum vitamin D [46]. No significant association between vitamin D status and BMI could be identified in our study, despite suggestions of association between these 2 parameters in the literature [[79], [80], [81]]. On average, in most of the included publications in our analysis, study populations were above the overweight threshold (BMI > 25), which may have limited our ability to detect an association with serum 25(OH)D levels.

Owing to the public health burden of widespread vitamin D deficiency, an appropriate response is necessary from public authorities. This seems especially important in women as they seem to have significantly lower serum vitamin D compared with men, which may have public health implications as vitamin D deficiency in women has been associated with a variety of outcomes related to pregnancy, such as miscarriage [9], preeclampsia, gestational diabetes [82], or fertility [10]. Regional initiatives have been implemented in the MENA region, such as wheat flour fortification, though mandatory only for iron and folic acid in most countries of the region. In recent years, however, KSA issued guidelines and recommendations related to food fortification with vitamin D, establishing mandatory fortification of wheat flour [83]. These initiatives, together with the fact that awareness of the issue has been raised thanks to an increasing number of scientific publications on the matter, may explain some trends toward decreased vitamin D deficiency prevalence in this country [84]. Other regional guidelines for vitamin D deficiency prevention have been issued, notably by the GCC countries, emphasizing safe sunlight exposure as the primary option for vitamin D deficiency prevention in the general population, followed by food fortification. Nutritional supplementation is also recommended in at-risk groups such as older women in these guidelines [70]. Despite this, our work still shows variability in the MENA region, and other countries may also benefit from the implementation of additional fortification policies or awareness campaigns [85].

Our study presents several limitations. First, the predominance of studies from KSA and Iran limits the extrapolation of the results to the whole region. Also, the pooled data were from a variety of studies with different protocols, notably, methods of measurement. Unfortunately, it was not possible to conduct a separate analysis with only studies using the reference standard for serum vitamin D measurement due to the limited number of studies. Standardization in future studies regarding the methods of measurement would help obtain more robust data. Additionally, our primary parameter being serum vitamin D, we had to exclude a significant number of studies (N = 9) with missing values.

In conclusion, our meta-analysis confirms widespread vitamin D deficiency in the MENA region despite abundant sunlight. The deficiency appears more pronounced in women than in men, whatever the age. These findings confirm the need for local strategies to prevent vitamin D deficiency, especially in women. Noting the heterogeneity between studies and the lack of data in several countries, more population-based studies are needed in the future to better inform populations depending on their specificities, habits, and environment.

## Author contributions

The authors’ responsibilities were as follows – RD, FM, LR, NB, MD-D: conceived the study; RD, LR, NB, MD-D: planned and designed the study; RD, NB, MD-D: designed the search strategy; LR, RD, MD-D: performed the searches; RD, LR: screened the titles, abstracts, and full texts of retrieved articles, with any disputes resolved in consultation with NB, MD-D; FM, RD: extracted data; RD: conducted risk of bias assessment, which was verified by NB and MD-D; RD, FM, LR, HB, MD-D: interpreted the data; FM, RD: drafted the manuscript; LR, RD, HB, JE, NB, MD-D: further contributed toward the writing and critical revision of the manuscript; and all authors: read and approved the final manuscript.

## Funding

Supported in part by Lesaffre International, Marcq-en-Baroeul, France. The sponsor had no role in the study design, data analysis, or interpretation of results.

## Conflict of interest

MD-D reports financial support and article publishing charges were provided by Lesaffre. FM, LR, JE, and MD-D report a relationship with Lesaffre that includes: employment. RD reports financial support and travel were provided by Lesaffre International Sarl. RD reports a relationship with Lesaffre International Sarl that includes: consulting or advisory. The other authors declare that they have no known competing financial interests or personal relationships that could have appeared to influence the work reported in this article.
