# Supplementary material for: Vitamin D Nutritional Status in the Middle East and North Africa Region: A Systematic Review and Meta-analysis
Source: Curr Dev Nutr. 2025 Oct 21;9(11):107577. doi: 10.1016/j.cdnut.2025.107577 (PMC12666523; doi:10.1016/j.cdnut.2025.107577)
Supplement: multimedia component 2 [file mmc2.pdf]

Supplementary Figures

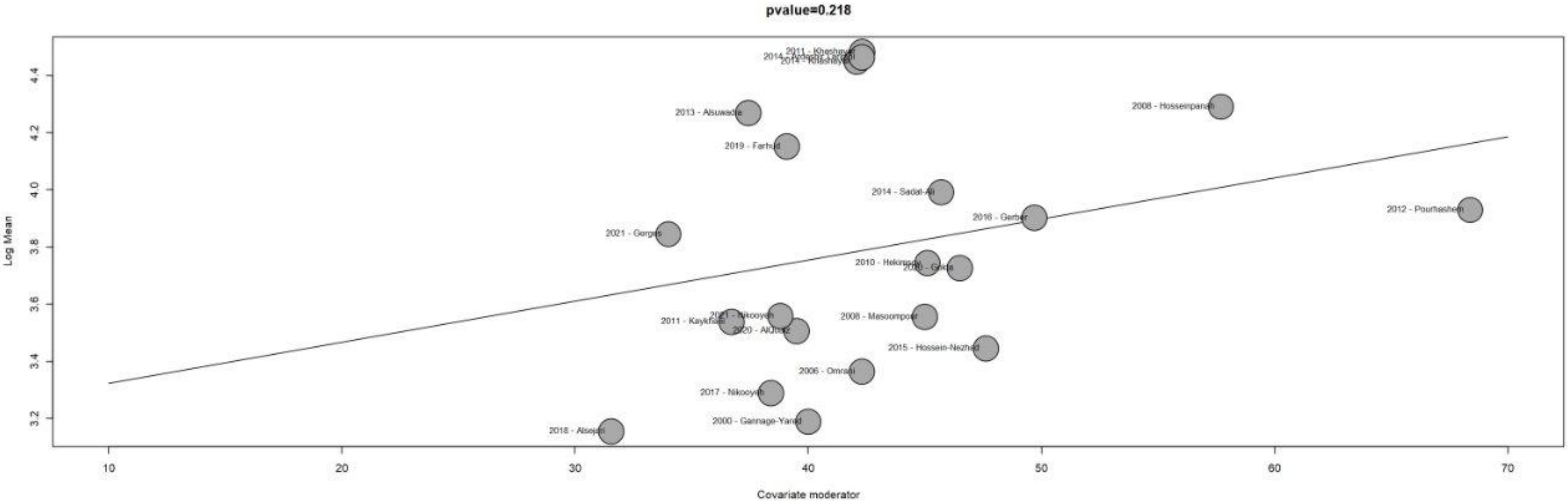

Baujat plot of the the impact of age on pooled serum vitamin D from included studies conducted in the Middle-East and North Africa (MENA) regions (N=21). The association was not significant.

Supplementary Figures

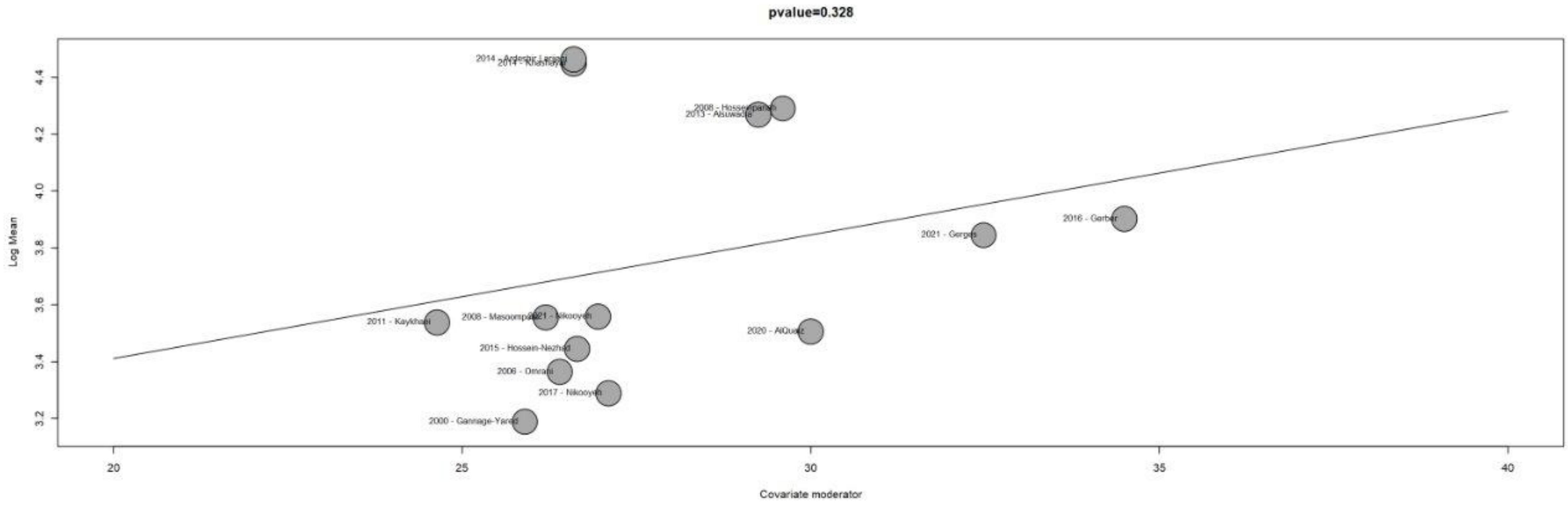

Baujat plot of the the impact of BMI on pooled serum vitamin D from included studies conducted in the Middle-East and North Africa (MENA) regions (N=14). The association was not significant.

Supplementary Figures

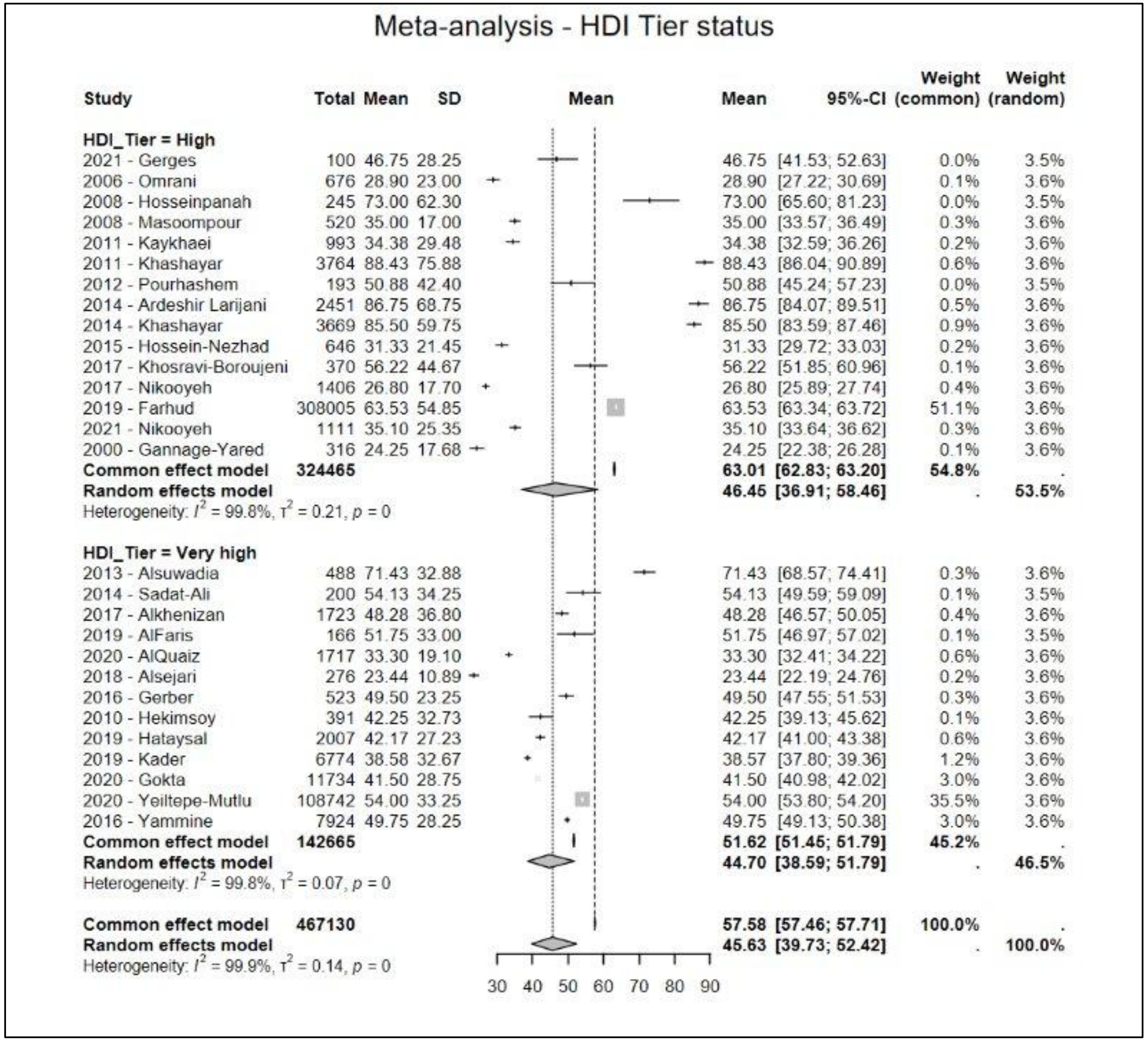

Forest plot of the impact of each country's Human Development Index (HDI) tier, on pooled serum vitamin D from included studies conducted in the Middle-East and North Africa (MENA) regions (N=28). The effect of HDI was not significant.

Supplementary Figures

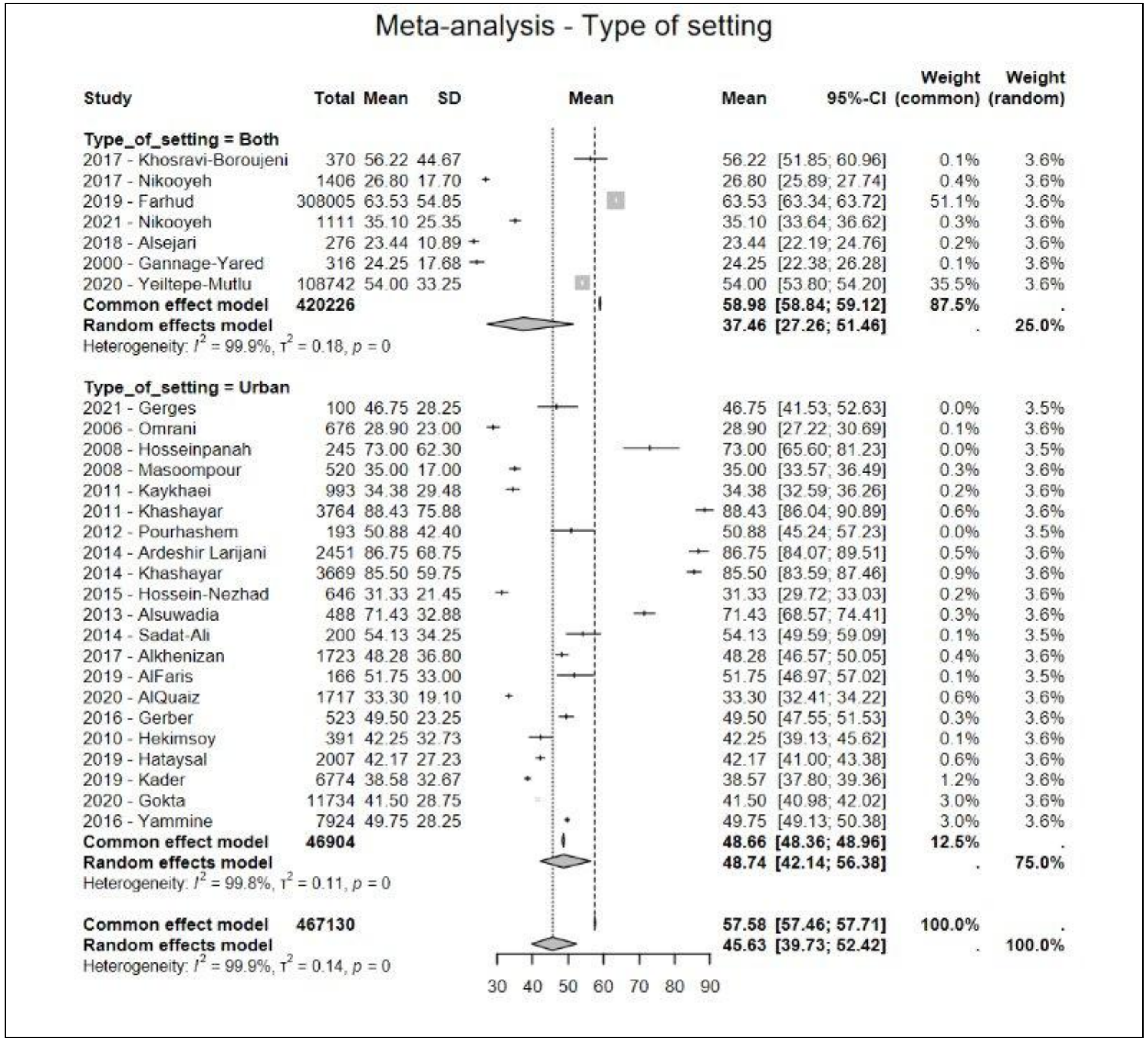

Forest plot of the impact of studies’ setting (urban, rural, or a mix of the two), on pooled serum vitamin D from included studies conducted in the Middle-East and North Africa (MENA) regions (N=28). The effect of setting was not significant.

Supplementary Figures

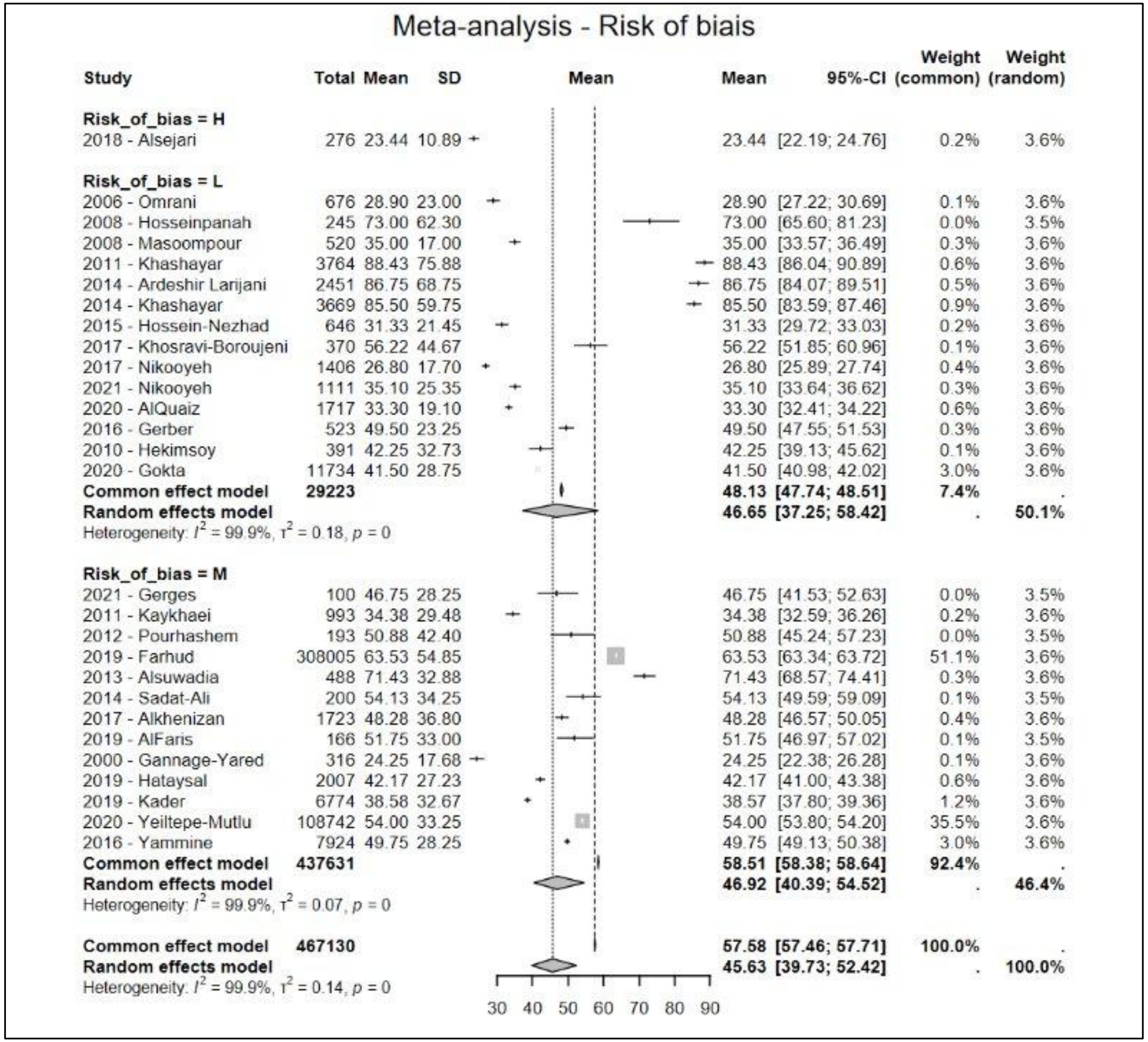

Forest plot of the impact of studies' risk of bias (low: L, medium: M, or high: H), on pooled serum vitamin D from included studies conducted in the Middle-East and North Africa (MENA) regions (N=28). No significant association were observed.
